# Interval changes in four-dimensional flow-derived in vivo hemodynamics stratify aortic growth in type B aortic dissection patients

**DOI:** 10.1016/j.jocmr.2024.101078

**Published:** 2024-08-02

**Authors:** Joshua Engel, Ozden Kilinc, Elizabeth Weiss, Justin Baraboo, Christopher Mehta, Andrew Hoel, S. Chris Malaisrie, Michael Markl, Bradley D. Allen

**Affiliations:** a Department of Radiology, Northwestern University, Chicago, Illinois, USA; b Department of Biomedical Engineering, Northwestern University, Chicago, Illinois, USA; c Department of Cardiac Surgery, Northwestern Medicine, Chicago, Illinois, USA; d Department of Vascular Surgery, Northwestern Medicine, Chicago, Illinois, USA

**Keywords:** Aortic dissection, 4D flow imaging, Flow, TEVAR

## Abstract

**Background:**

Aortic diameter growth in type B aortic dissection (TBAD) is associated with progressive aortic dilation, resulting in increased mortality in patients with both de novo TBAD (dnTBAD) and residual dissection after type A dissection repair (rTAAD). Preemptive thoracic endovascular aortic repair may improve mortality in patients with TBAD, although it is unclear which patients may benefit most from early intervention. In vivo hemodynamic assessment using four-dimensional (4D) flow cardiovascular magnetic resonance (CMR) has been used to characterize TBAD patients with growing aortas. In this longitudinal study, we investigated whether changes over time in 4D flow-derived true and false lumen (TL and FL) hemodynamic parameters correlate with aortic growth rate, which is a marker of increased risk.

**Methods:**

We retrospectively identified TBAD patients with baseline and follow-up 4D flow CMR at least 120 days apart. Patients with TBAD intervention before baseline or between scans were excluded. 4D flow CMR data analysis included segmentation of the TL and FL, followed by voxel-wise calculation of TL and FL total kinetic energy (KE), maximum velocity (MV), mean forward flow (FF), and mean reverse flow (RF). Changes over time (Δ) were calculated for all hemodynamic parameters. Maximal diameter in the descending aorta was measured from magnetic resonance angiogram images acquired at the time of 4D flow. Aortic growth rate was defined as the change in diameter divided by baseline diameter and standardized to scan interval.

**Results:**

Thirty-two patients met inclusion criteria (age: 56.9 ± 14.1 years, female: 13, n = 19 rTAAD, n = 13 dnTBAD). Mean follow-up time was 538 days (range: 135–1689). Baseline aortic diameter did not correlate with growth rate. In the entire cohort, Δ FL MV (Spearman’s rho [rho] = 0.37, p = 0.04) and Δ FL RF (rho = 0.45, p = 0.01) correlated with growth rate. In rTAAD only, Δ FL MV (rho = 0.48, p = 0.04) and Δ FL RF (rho = 0.51, p = 0.03) correlated with growth rate, while in dnTBAD only, Δ TL KE (rho = 0.63, p = 0.02) and Δ TL MV (rho = 0.69, p = 0.01) correlated with growth rate.

**Conclusion:**

4D flow-derived longitudinal hemodynamic changes correlate with aortic growth rate in TBAD and may provide additional prognostic value for risk stratification. 4D flow MRI could be integrated into existing imaging protocols to allow for the identification of TBAD patients who would benefit from preemptive surgical or endovascular intervention.

## Graphical abstract

**Figure:**
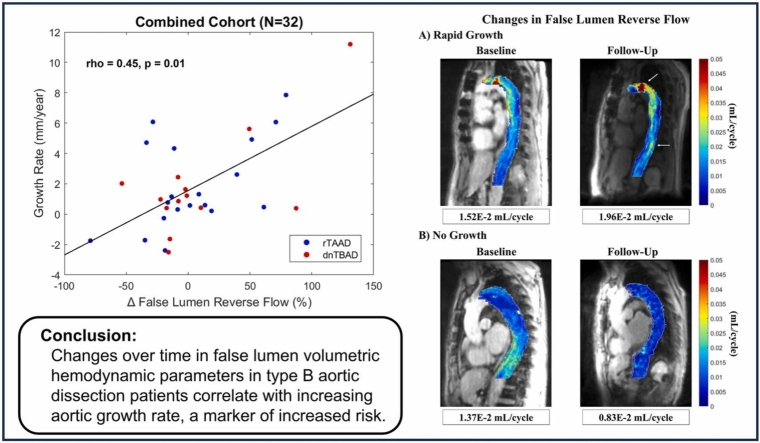

## 1 Introduction

Aortic dissection (AD) is caused by a tear in the aortic intima, resulting in two parallel channels of blood flow in the aorta known as the true lumen (TL) and false lumen (FL). AD is classified into Stanford type A aortic dissection (TAAD) if it originates in the ascending aorta or Stanford type B aortic dissection (TBAD) if it originates distal to the left subclavian artery in the descending aorta. TBAD can be further divided into de novo TBAD (dnTBAD), in which the primary entry tear and dissection are contained in the descending aorta, or repaired TAAD (rTAAD), in which there is unresolved dissection extending into the descending aorta following surgical ascending aorta intervention in TAAD [1], [2], [3], [4]. In the absence of intervention, all types of TBAD transition to a chronic state as the aorta remodels [5], [6]. However, there remains a risk for continued growth in the descending aorta and aneurysmal expansion in the chronic state, which can lead to complications including end-organ malperfusion, rupture, and death. Considering this, chronic TBAD still carries significant mortality, with 3-year mortality rates approaching 25% [7].

Even with optimized heart rate and blood pressure control, 20–50% of medically managed chronic TBAD patients still require surgical intervention at some point in their clinical course, often due to rapid aortic expansion or aneurysmal degeneration, with larger aortic diameters associated with increased risk of rupture or end-organ malperfusion [8], [9], [10]. Because of this risk, routine computed tomography angiography or magnetic resonance angiography (MRA) is recommended for chronic TBAD patients at 1, 3, 6, and 12 months following the dissection event and then annually if stable [4], [11], [12].

Improving preemptive management and risk stratification of TBAD is of particular interest given these persistently high adverse outcome rates. There is evidence that preemptive thoracic endovascular aortic repair (TEVAR) in chronic TBAD improves all-cause 3-year mortality [13]. However, it is unclear which subgroups of TBAD patients are most likely to benefit from early intervention, as TEVAR still carries its own morbidity risk. Imaging-based risk stratification for late complications of TBAD has historically been confined to the evaluation of morphologic information, including baseline maximum descending aortic diameter and aortic growth rate [4], [7], [14]. Four-dimensional (4D) flow cardiovascular magnetic resonance (CMR) can be used to noninvasively capture the complex hemodynamics within AD, and in vivo hemodynamic assessment may help characterize TBAD patients with unstable and enlarging aortas [2], [3], [15], [16], [17]. Moreover, a recent study showed that 4D flow-derived voxel-wise hemodynamic parameters in the TL and FL at baseline correlated with later need for aortic surgery or aorta-related death and aortic growth in TBAD patients [1]. In this longitudinal study, we expand on these findings by tracking changes in voxel-wise hemodynamic parameters over time in serial 4D flow CMR scans. We hypothesize that changes over time in TL and FL hemodynamic parameters will correlate with increasing aortic growth rate in TBAD patients.

## 2 Methods

### 2.1 Study cohort

This study was performed in accordance with two Institutional Review Board (IRB)-approved protocols. A portion of the subjects were included under a retrospective IRB protocol with the waiver of informed consent and had 4D flow CMR included in their standard of care clinical imaging. The remaining subjects were prospectively identified and provided signed informed consent to 4D flow CMR in addition to their standard of care clinical imaging. All patients were recruited at a large tertiary center. Inclusion criteria are as follows: known dnTBAD or rTAAD with ≥120 days of 4D flow imaging follow-up between baseline and follow-up scans. Patients with prior descending aorta surgical or endovascular intervention or intervention between scan dates were excluded.

### 2.2 Image acquisition

All images were acquired using 1.5T MR-systems (Magneton Avanto, Aera, or Sola, Siemens Healthineers, Erlangen, Germany). 4D flow CMR data captured before 2020 used prospective electrocardiogram (ECG) cardiac triggering and respiratory navigators in a sagittal oblique orientation. Scans captured from 2020 onward used retrospective ECG gating without respiratory navigator gating in a coronal orientation. Protocol changes were a result of institutional augmentations to clinical 4D flow implementation. Scan parameters for prospective scans were as follows: spatial resolution = 2.5 mm^3^, field of view (FOV) = 255–365 × 340–450 mm^2^, slab thickness = 28–50 mm, temporal resolution = 36.8–65.6 ms, repetition time (TR) = 5.3–9.4 ms, echo time (TE) = 2.2–2.5 ms, flip angle = 7–15°, and velocity sensitivity (venc) = 160 cm/s. Scan parameters for retrospective scans were as follows: spatial resolution = 2.5 mm^3^, FOV = 285–407 × 380–459 mm^2^, slab thickness = 25–40 mm, temporal resolution = 22.7–54.0 ms, TR = 4.9–5.8 ms, TE = 2.0– 3.0 ms, flip angle = 7–15° and venc = 160 cm/s.

### 2.3 Image processing and segmentation

**Fig. 1:**
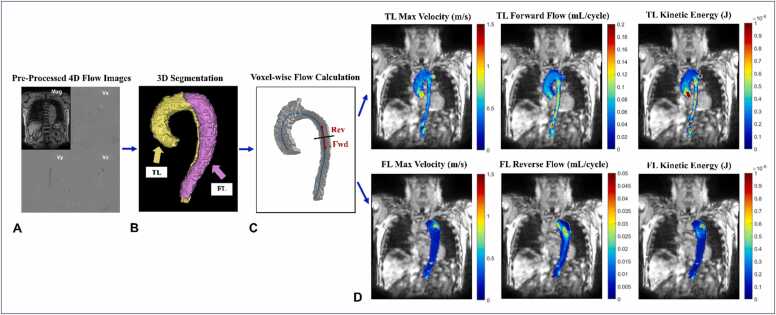
4D flow MRI pre- and post-processing workflow. (A) Eddy current correction, noise-masking of areas outside of flow regions, and velocity anti-aliasing. (B) Manual 3D aortic segmentation of the true and false lumens. (C) Automatic calculation of 3D centerline along the path of blood flow in the TL for voxel-wise definition of flow direction based on the closest orthogonal plane. (D) Hemodynamic maps in the TL (top) and FL (bottom). *TL* true lumen, *FL* false lumen, *Rev* reverse, *Fwd* forward, *4D* four-dimensional, *3D* three-dimensional.

A diagram of the image processing workflow and parametric maps is shown in Fig. 1. 4D flow CMR images were pre-processed using eddy current correction, noise-masking of areas outside of flow regions, and velocity anti-aliasing using a home-built tool (MATLAB; MathWorks, Natick, Massachusetts, USA) [18]. Time-averaged magnitude and three-dimensional (3D) phase-contrast angiogram images were generated following image pre-processing. Time-averaged magnitude images were used to manually segment the entire aorta (TL+FL) from the level of the aortic valve to the level of the celiac artery, excluding all vessels arising from the aortic arch. The 3D phase-contrast angiogram images calculated from the mean sum of squares of velocity data were used to segment the TL. The FL segmentations were generated by subtraction of the TL from whole aorta segmentations. Each patient’s aorta was segmented twice, with one segmentation for the baseline scan and one for the follow-up scan. Two TL regions in the proximal and distal descending thoracic aorta (DAo) were generated by placing planes between the left common carotid and subclavian arteries and in the DAo at the level of the superior pulmonary veins. Two FL regions, proximal and distal, were generated by qualitatively placing a plane that bisected the length of the whole FL segmentation. All manual segmentations were performed by two observers (J.S.E. and O.K.) using designated image segmentation software (Mimics Innovation Suite; Materialise, Leuven, Belgium).

### 2.4 Parametric hemodynamic maps

3D parametric maps of aortic hemodynamics were generated using an in-house analysis tool (MATLAB; MathWorks, Natick, Massachusetts) similar to a recently reported workflow [2]. The 4D flow velocity data were interpolated to 1 mm^3^ using spline interpolation. For each voxel inside both the TL and the FL, kinetic energy (KE), forward flow (FF), reverse flow (RF), maximum velocity (MV), and viscous energy loss (EL) were calculated. Changes over time (Δ) in all parameters between baseline and follow-up scans were calculated. Because our study cohort included both retrospectively gated and prospectively triggered scans, the minimum % cardiac cycle imaged in any single prospective scan (67.3%) was used as a cutoff for the entire cohort when creating parametric hemodynamic maps and calculating aortic voxel-wise and volumetric sums. These percentages were calculated using each patient’s respective heart rate during each 4D flow CMR scan.

### 2.5 Forward flow and reverse flow

The TL segmentation was used to generate a 3D aortic centerline along the path of TL flow, with orthogonal analysis planes placed every millimeter along the centerline. The direction of FF for both the TL and FL was set by the normal vector of each plane along the length of the aortic centerline. Each voxel was matched to the nearest plane to determine forward or RF at each time point. FF and RF were reported as means and calculated by summing flow at each voxel over the cardiac cycle, then averaging the sums over the entire luminal volume.

### 2.6 Maximum velocity

The time point with the highest 95th percentile voxel-wise MV was used to create the 3D MV maps. The time points used for the TL and FL were selected independently. The mean of the maximum top 5% of velocities is reported as MV for the TL and FL.

### 2.7 Kinetic energy

Voxel-wise KE was calculated using the following equation:

$$\mathrm{KE}=0.5\times \rho \times \mathrm{dV}\times {v(t)}^{2}$$

With ρ as the density of blood assumed as 1060 kg/m^3^ and dV as the unit voxel volume. Reported KE was calculated as total luminal KE by summing each voxel over the cardiac cycle and then over the entire luminal volume.

### 2.8 Energy loss

Viscous energy dissipation (ϕ_v_) was calculated at each voxel over time using the following equation [19]:

$$\phi_{v}=\frac{1}{2}\sum_{i}\sum_{j}{\left[\left(\frac{{\partial v}_{j}}{{\partial x}_{i}}+\frac{{\partial v}_{i}}{{\partial x}_{j}}\right)-\frac{2}{3}\left(\nabla \cdot V\right)\delta_{\mathrm{ij}}\right]}^{2}$$

where $\delta_{\mathrm{ij}}$ = 1 for *i* = *j*; $\delta_{\mathrm{ij}}$ = 0 for *i ≠ j*.

Viscous energy dissipation was then multiplied by voxel volume and blood viscosity to measure the voxel-wise EL rate. This was integrated over time for each voxel to measure total EL (EL_total_), and the mean across all voxels for each region was reported. EL was also averaged over time for each voxel to measure mean EL rate (EL_mean_), and the mean across all voxels for each region was reported. Blood was assumed to be Newtonian and incompressible with a density of 1060 kg/m^3^ and viscosity of 3.2 cP. EL was calculated for the FL and the combined DAo, comprised of the FL and the TL from the level of the left subclavian artery to the distal thoracic aorta.

### 2.9 Distal anastomotic entry tears, morphologic measurements, and aorta growth rate

For each subject, standard high-resolution MRA images were included as part of their CMR protocol. Distal anastomotic new entry tears (DANE) in rTAAD patients were detected based on the presence of flow jets visualized on baseline 4D flow imaging (cvi42, Circle Cardiovascular Imaging, Calgary, Alberta, Canada). DANE location within the distal arch or proximal DAo was verified by overlaying 3D velocity maximal intensity projections onto anatomical images from 4D flow acquisitions. Maximal dissection diameter, proximal entry tear diameter, distance of proximal entry tears to the left subclavian artery, and FL thrombosis level at baseline were determined using dedicated visualization and multiplanar reformation software (Visage 7, Visage Imaging, Inc., San Diego, California). Maximal dissection diameter in the descending aorta, across both the TL and FL, was determined with the multiplanar double oblique method. All diameter measurements were performed by the same observer (J.S.E.), trained by an experienced cardiovascular radiologist (B.D.A.). All other morphological measurements were made by B.D.A. Aortic growth rate was defined as the difference in total aortic diameter between scans divided by the time interval between scans, expressed as mm/year. Aortic growth rate was used as an outcome because of its historical use as a marker of aortic instability, the increasing rate of aortic rupture at larger diameters, and indications for surgical intervention based on total aortic diameter and growth rate [6], [10], [11], [16], [20]. Rapid aortic growth was defined as greater than 3 mm/year [6], [20].

## 3 Statistical analysis

Shapiro-Wilk normality tests and Q-Q plots were used to assess the distribution of the data. For all groupwise comparisons, independent T-tests and Mann-Whitney U-tests were used for normally distributed data and non-normally distributed data, respectively. For groupwise comparisons of categorical demographic parameters, a chi-square analysis was used. For analysis of entry tears, patients in whom an entry tear could not be visualized were excluded. Spearman correlations were performed between scan-interval normalized changes in hemodynamic parameters, baseline hemodynamic parameters, baseline aortic diameter, proximal entry tear size, distance from the proximal entry tear to the left subclavian artery, and aortic growth rate. For analysis of DANE, a 1-tailed U-test was used with the hypothesis that patients with DANE displayed faster aortic growth. The statistical significance level was set as alpha = 0.05.

## 4 Results

### 4.1 Patient demographics

**Fig. 2:**
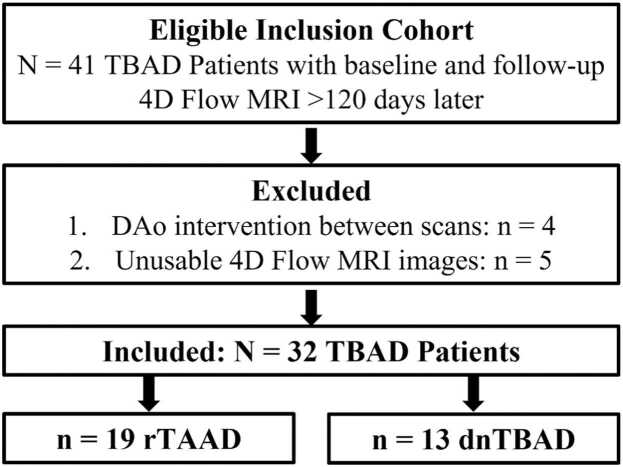
Cohort selection flowchart. The eligible inclusion cohort included retrospectively identified and prospectively enrolled TBAD patients. *TBAD* type B aortic dissection, *rTAAD* repaired type A aortic dissection, *dnTBAD* de novo TBAD, *4D* four-dimensional, *MRI* magnetic resonance imaging, *DAo* descending thoracic aorta.

A total of n = 41 TBAD patients were identified with baseline and follow-up 4D flow CMR. Of these 41 patients, n = 4 patients were excluded due to descending aorta intervention between scans and n = 5 patients were excluded due to 4D flow data being unusable as a result of missing slices or unacceptable spatial aliasing artifact (Fig. 2). After this, n = 32 patients were included in the final cohort, of which 22 were identified retrospectively and 10 were enrolled prospectively. The mean age was 56.9 ± 14.1 years and there were 19 males and 13 females. Within the cohort, there were n = 19 rTAAD and n = 13 dnTBAD cases. The baseline hemodynamic parameters and aortic growth rates of n = 30 patients in this cohort (94%) were reported in a previously published study [1]. The median interval between 4D flow scans was 215 days with an interquartile range (IQR) of 685 days, and of these scans, n = 24 were prospectively gated and n = 40 were retrospectively gated. In the whole cohort, the median time from presentation to baseline 4D flow was 1.29 years with an IQR of 1.95 years and the median total clinical follow-up time from presentation was 6.44 years with an IQR of 4.63 years. There were n = 3 patients who went on to have a descending aorta intervention after the second 4D flow scan, consisting of n = 1 frozen elephant trunk procedure, n = 1 elephant trunk with extension TEVAR, and n = 1 with descending aorta graft repair, all of which had prior TAAD repair.

Comparison of demographics and morphologic parameters between patients with rTAAD and dnTBAD are listed in Table 1. Proximal entry tears were identified in 24 out of 32 patients, of which 14 were in the rTAAD subgroup and 10 were in the dnTBAD subgroup. Patients in the rTAAD subgroup were more often in the chronic TBAD phase upon presentation for baseline 4D flow imaging, whereas more patients in the dnTBAD group had baseline 4D flow scans during the acute or subacute phases of TBAD. No other demographic or morphologic factors significantly differed between patients with rTAAD and dnTBAD (p > 0.05).

**Table 1:** Comparison of demographic and morphologic factors in the cohort broken into rTAAD and dnTBAD.

Parameter			Repaired TAAD	De novo TBAD	p-value
Age (years)		Average	58.3 ± 15.8	58.4 ± 12.6	1.00
BMI			26.6 ± 5.84	28.0 ± 7.6	0.56
Baseline systolic blood pressure (mmHg)			124.8 ± 13.6	124.7 ± 10.4	0.97
Baseline pulse pressure (mmHg)			56.2 ± 14.6	53.3 ± 8.0	0.48
Follow-up systolic blood pressure (mmHg)			122.4 ± 10.9	130.8 ± 20.7	0.20
Follow-up pulse pressure (mmHg)			53.9 ± 12.2	53.0 ± 11.7	0.83
Baseline heart rate (bpm)			66.8 ± 10.4	72.0 ± 8.6	0.14
Scan interval (years)			0.59 (1.79)	0.58 (1.98)	0.73
Baseline diameter (mm)			45.9 ± 7.2	44.5 ± 4.8	0.57
Entry tear diameter (mm)			6.0 (9.0)	7.0 (6.3)	0.41
				
Male sex		n (%)	13 (68.4)	6 (46.2)	0.21
Acuity	Acute		0 (0)	3 (23.1)	**0.03**
	Subacute		1 (5.3)	2 (15.4)	0.33
	Chronic		18 (94.7)	8 (61.5)	**0.02**
Positive smoking history			9 (47.4)	6 (46.2)	0.95
Connective tissue disease			3 (15.8)	3 (23.1)	0.60
Medications	Anti-hypertensive		19 (100)	13 (100)	1.00
	Aspirin		14 (73.7)	8 (61.5)	0.47
	Statin		15 (78.9)	8 (61.5)	0.28
	Warfarin		4 (21.1)	0 (0)	0.08
Entry tear location	Aortic arch		9	5	-
	Proximal DAo		4	1	-
	Distal DAo		1	4	-
FL thrombosis percentage	>25% Thrombus		7 (36.8)	5 (38.5)	0.93
	<25% Thrombus		12 (63.2)	8 (61.5)	0.93

*rTAAD* repaired type A aortic dissection*, dnTBAD* de novo type B aortic dissection*, TAAD* type A aortic dissection*, TBAD* type B aortic dissection*, BMI* body mass index*, DAo* descending thoracic aorta*, FL* false lumen*, 4D* four-dimensional*, SD* standard deviation*, IQR* interquartile range*.*

No demographic parameters differed significantly between the rTAAD and dnTBAD subgroups, except a greater proportion of the rTAAD subgroup was in the chronic TBAD phase upon presentation for baseline 4D flow imaging and more patients in the dnTBAD group had baseline 4D flow scans during the acute or subacute phases of TBAD. Bold indicates significance.

Baseline diameter is the maximum diameter across both the true and false lumens in the descending thoracic aorta. In the top section of the table, variables are either reported as mean ± SD if normally distributed or median (IQR) if not.

### 4.2 Overall cohort

**Fig. 3:**
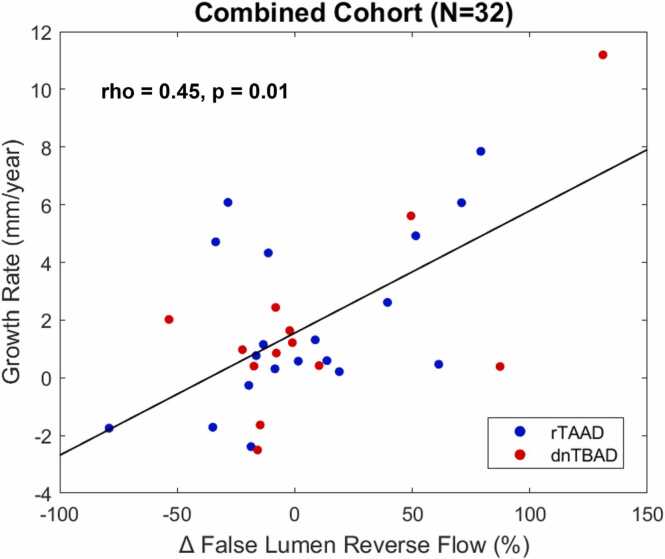
Scatter plot showing the relationship between change in FL RF and aortic growth rate. *rTAAD* repaired type A aortic dissection, *dnTBAD* de novo type B aortic dissection, *rho* Spearman’s rho, *FL* false lumen, *RF* reverse flow.

**Fig. 4:**
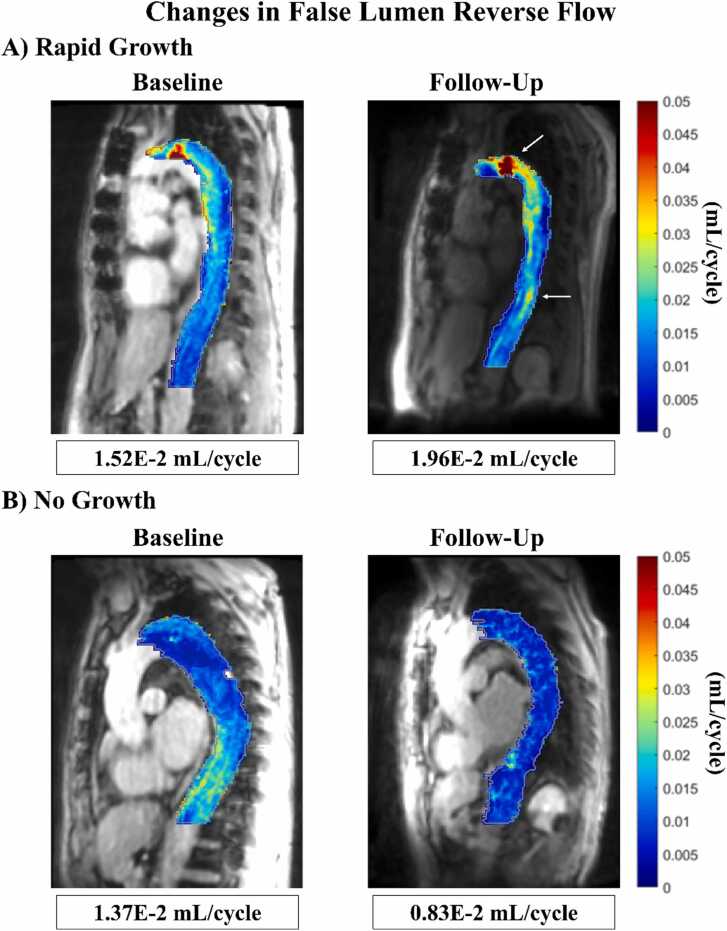
Parametric maps of false lumen reverse flow at baseline and follow-up in (A) a patient with a growth rate of 7.8 mm/year, captured 135 days apart. Arrows point to areas of increased reverse flow in the early descending aorta near the primary entry tear and in the distal descending aorta. (B) A patient with a growth rate of −2.5 mm/year, with scans captured 2.48 years apart.

The overall mean change in total aortic diameter was 2 ± 3 mm, with a median growth rate of 1.1 mm/year and IQR of 2.7 mm/year. Δ FL MV (Spearman’s rho [rho] = 0.37, p = 0.04) and Δ FL RF (rho = 0.45, p = 0.01) positively correlated with aortic growth rate (Table 2, Fig. 3). There was a trend toward increases in TL MV correlating with aortic growth rate, but it did not meet the significance level (rho = 0.35, p = 0.051). Baseline levels of the six hemodynamic parameters studied did not correlate with aortic growth rate in this cohort, nor did baseline aortic diameter (rho = −0.15, p = 0.41) ( Tables 3 and 4). An example of FL RF maps showing increased RF in the early descending aorta near the primary entry tear and in the distal descending aorta for a patient with rapid aortic growth is shown in Fig. 4 and compared to FL RF maps for a patient with no aortic growth. There were no significant correlations between baseline EL or Δ EL and aortic growth rate for either the FL or combined DAo. EL_total_ and EL_mean_ are reported in Supplementary Table 1. There were no significant correlations between hemodynamic parameters and changes in the segmented TL and FL volumes. Proximal entry tear diameter, distance of the proximal entry tear to the left subclavian artery, and FL thrombosis status were not significantly correlated with aortic growth rate (Table 4).

**Table 2:** Correlations between changes in hemodynamic parameters and aortic growth broken into overall cohort, rTAAD, and dnTBAD.

Overall cohort (N = 32)				Repaired TAAD only (n = 19)			De novo TBAD only (n = 13)		
Change in parameter (%)		Rho	p-value		Rho	p-value		Rho	p-value
TL KE	−4.94 (24.3)	0.30	0.10	−4.30 (25.4)	0.17	0.49	−5.58 (21.1)	0.63	**0.02**
TL MV	−2.09 (14.3)	0.35	0.051	−1.18 (11.3)	0.13	0.59	−3.54 (18.3)	0.69	**0.01**
TL FF	−2.30 (16.6)	0.10	0.60	−3.79 (20.6)	−0.15	0.55	−0.82 (9.85)	0.40	0.17
FL KE	−18.1 (46.1)	0.28	0.12	−15.1 (68.1)	0.21	0.39	−21.1 (43.4)	0.21	0.48
FL MV	−6.42 (27.6)	0.37	**0.04**	−6.61 (29.6)	0.48	**0.04**	−3.04 (19.9)	0.18	0.57
FL RF	−8.01 (41.8)	0.45	**0.01**	−8.45 (48.3)	0.51	**0.03**	−7.86 (26.2)	0.29	0.34

*rTAAD* repaired type A aortic dissection*, dnTBAD* de novo type B aortic dissection*, TAAD* type A aortic dissection*, TL* true lumen*, FL* false lumen*, KE* kinetic energy*, MV* maximum velocity*, FF* forward flow*, RF* reverse flow*, Rho* Spearman’s rho*, IQR* interquartile range*.*

Variables are reported as median (IQR). Bold indicates significance.

**Table 3:** Hemodynamic parameter values for baseline and follow-up scans with correlations with growth rate broken into overall cohort, rTAAD, and dnTBAD.

Overall cohort (N = 32)				Repaired TAAD only (n = 19)			De novo TBAD only (n = 13)		
Baseline parameter value		Rho	P-value		Rho	P-value		Rho	P-value
TL KE (J)	2.51E−1 ± 1.83E−1	−0.21	0.26	2.83E−1 ± 2.12E−1	−0.51	**0.03**	2.03E−1 ± 1.22E−1	0.42	0.15
TL MV (m/s)	1.36 ± 0.379	−0.13	0.47	1.44 ± 0.421	−0.34	0.16	1.24 ± 0.285	0.23	0.46
TL FF (mL/cycle)	1.12E−1 ± 3.42E−2	−0.14	0.46	1.21E−1 ± 3.82E−2	−0.30	0.22	1.11E−1 ± 2.76E−2	0.20	0.51
FL KE (J)	2.82E−2 ± 1.87E−2	0.14	0.46	2.63E−2 ± 1.64 ± −2	0.061	0.80	3.10E−2 ± 2.21E−2	0.21	0.49
FL MV (m/s)	4.30E−1 ± 1.61E−1	0.12	0.52	3.82E−1 ± 1.44E−1	−0.014	0.96	4.99E−1 ± 1.64E−1	0.27	0.36
FL RF (mL/cycle)	1.52E−2 ± 6.35E−3	−0.16	0.39	1.61E−2 ± 7.02E−3	−0.28	0.24	1.38E−2 ± 5.2E−3	0.082	0.79
					
Follow-up parameter value									
TL KE (J)	2.26E−1 ± 1.37E−1	-	-	2.34E−1 ± 1.37E−1	-	-	2.13E−1 ± 1.41E−1	-	-
TL MV (m/s)	1.33 ± 0.386	-	-	1.41 ± 0.386	-	-	1.20 ± 0.356	-	-
TL FF (mL/cycle)	1.14E−1 ± 3.33E−2	-	-	1.17E−1 ± 3.22E−2	-	-	1.10E−1 ± 3.56E−2	-	-
FL KE (J)	2.79E−2 ± 2.54E−2	-	-	2.62E−2 ± 2.40E−2	-	-	3.03E−2 ± 2.81E−2	-	-
FL MV (m/s)	4.23E−1 ± 1.85E−1	-	-	3.99E−1 ± 1.94E−1	-	-	4.59E−1 ± 1.72E−1	-	-
FL RF (mL/cycle)	1.60E−2 ± 7.53E−3	-	-	1.71E−2 ± 8.77E−3	-	-	1.45E−2 ± 5.16E−3	-	-

*rTAAD* repaired type A aortic dissection*, dnTBAD* de novo type B aortic dissection*, TAAD* type A aortic dissection*, TL* true lumen*, FL* false lumen*, KE* kinetic energy*, MV* maximum velocity*, FF* forward flow*, RF* reverse flow*, Rho* Spearman’s rho*, SD* standard deviation*.*

Kinetic energy is reported as the sum across the luminal volume. FF and RF are reported as mean values averaged over the luminal volume. Variables are reported as mean ± SD. Bold indicates significance.

**Table 4:** Relationships between selected morphological parameters and aortic growth rate.

Overall cohort (N = 32)			
*Morphologic parameter*		*Rho*	*P-value*
Baseline diameter (mm)	45.3 ± 6.3	−0.15	0.41
Entry tear diameter (mm)	9 (3.3)	0.34	0.10
ET distance to L subclavian artery (mm)	16 (53]	−0.27	0.26
	Aortic growth rate (mm/year)		
	*>25% Thrombus (n* *=* *12*)	*<25% Thrombus (n* *=* *20)*	*P-value*
FL thrombosis status	0.4 (2.8)	1.2 (2.6)	0.22

Repaired TAAD only (n = 19)			
	Aortic growth rate (mm/year)		
	*DANE present (n* *=* *10)*	*No DANE present (n* *=* *9)*	
Presence of DANE	1.9 (4.3)	0.2 (3.0)	**0.04**

*ET* entry tear*, L* left*, FL* false lumen*, TAAD* type A aortic dissection*, DANE* distal anastomotic new entry tear*, Rho* Spearman’s rho*, SD* standard deviation*, IQR* interquartile range*.*

Variables are either reported as mean ± SD if normally distributed or median (IQR) if not. Bold indicates significance.

### 4.3 Repaired TAAD

In the n = 19 rTAAD patients, the mean change in total aortic diameter was 2 ± 2 mm. The median growth rate in this subgroup was 1.2 mm/year with an IQR of 4.3 mm/year. Δ FL MV (rho = 0.48, p = 0.04) and Δ FL RF (rho = 0.51, p = 0.03) positively correlated with growth rate (Table 2). Baseline TL KE significantly negatively correlated with aortic growth rate (rho = −0.51, p = 0.03), but other baseline levels of hemodynamic parameters and baseline aortic diameter did not significantly correlate with aortic growth rate (Table 3). rTAAD patients with DANE had higher aortic growth rates on average than those without (median 1.9 mm/year, IQR 4.3 vs median 0.2 mm/year, IQR 3.0, p-value = 0.04). Between subgroups, the degree of changes in hemodynamic parameters was not significantly different.

### 4.4 De novo TBAD

In the n = 13 dnTBAD patients, the mean change in total aortic diameter was 2 ± 3 mm, with a median growth rate of 1.0 mm/year with an IQR of 1.6 mm/year. In this subgroup, Δ TL KE (rho = 0.63, p = 0.02) and Δ TL MV (rho = 0.69, p = 0.01) correlated positively with aortic growth rate (Table 2). In patients with dnTBAD, systolic blood pressure at follow-up was similar to baseline systolic blood pressure (124.7 vs 130.8 mmHg, p = 0.34). Systolic blood pressure at follow-up did not correlate with growth rate (p = 0.77). Baseline levels of hemodynamic parameters and baseline aortic diameter did not significantly correlate with aortic growth rate (Table 3).

### 4.5 Regional analysis

In the overall cohort, Δ KE in the proximal TL (rho = 0.36, p = 0.046), Δ KE in the distal TL (rho = 0.36, p = 0.045), and Δ FF in the distal TL (rho = 0.39, p = 0.03) positively correlated with aortic growth rate. In the dnTBAD subgroup, Δ FF in the proximal TL (rho = 0.60, p = 0.03) positively correlated with aortic growth rate. There were no significant correlations between changes in regional FL hemodynamics or hemodynamic values at baseline and aortic growth rate.

## 5 Discussion

In this longitudinal study assessing changes in 4D flow CMR-derived hemodynamic parameters, we found that changes in multiple parameters over time correlated with aortic growth rate in patients with AD. In the combined cohort of both rTAAD and dnTBAD patients, increases in Δ FL MV and Δ FL RF correlated with aortic growth. In the subgroup of only rTAAD patients, the relationships between FL MV and FL RF and growth were significant and patients with DANE had higher aortic growth rates; however, the subgroup of only dnTBAD patients displayed different results. In dnTBAD only, increases in TL KE and TL MV correlated with growth, whereas the correlations with Δ FL MV and Δ FL RF were not statistically significant. Across the cohort, baseline maximal aortic diameter was not predictive of growth, nor were proximal entry tear diameter, distance of the proximal entry tear to the left subclavian artery, and FL thrombosis level [21]. Notably, relative to our prior work assessing interobserver variability (see Supplementary Table 2 in Chu et al.), the magnitudes of hemodynamic changes over time are larger than expected for interobserver variability. This suggests that the observed changes are real and unlikely due to the data analysis approach [1].

There is a growing body of evidence supporting the use of 4D flow CMR, and more broadly, hemodynamic assessment, in evaluation of aortic growth and risk stratification in TBAD. Recent studies have demonstrated that FL hemodynamics may play a role in the pathogenesis of aortic growth and poor clinical outcomes in patients with chronic TBAD. Chu et al. showed that patients with higher levels of KE in the FL relative to KE in the TL using 4D flow CMR went on to have greater aortic growth [1]. Allen et al. found that higher FL ejection fraction, which is the ratio of retrograde flow to antegrade flow at the primary entry tear, was associated with adverse aorta-related outcomes, and other studies have shown that FL ejection fraction and pressurization correlate with aortic growth [15], [16], [17]. And lastly, Evangelista et al. showed that in a longitudinal cohort study of 131 patients with TBAD, higher levels of systolic antegrade FL flow and diastolic retrograde FL flow were independent predictors of both aortic growth and adverse clinical outcomes [22].

Our study expanded on these findings by investigating changes in FL hemodynamics longitudinally in a heterogeneous cohort of patients with TBAD. Our findings that increases in FL MV and RF correlated with aortic growth in the combined cohort and that the presence of DANE in rTAAD patients, which has been linked to increased rates of adverse outcomes and patent FL, was associated with higher aortic growth rates build upon the conclusions of these prior studies [23]. Importantly, our study utilized flow jets between TL and FL to identify DANE as opposed to anatomical evaluation [24]. These results may be pointing to dynamism in a patent FL as a driver of aortic expansion, in that increases in high velocity, complex flow may be driving FL expansion. FL RF, which often extends into diastole and can signify the presence of distal re-entry tears or emptying of FL blood volume through proximal tears, may capture the ejection of blood from the FL due to pressurization and inhibition of antegrade flow and would be in line with results of studies relating FL ejection fraction and diastolic planar FL retrograde flow to aortic growth rate [15], [16], [17], [22].

Other studies have investigated measures of flow turbulence and vorticity in TBAD. Ruiz-Muñoz et al. found that FL in-plane rotational flow positively correlated with aortic growth rate, hypothesizing it as a marker of inhibition of antegrade flow and FL pressurization [25]. However, they did not find an association between FL retrograde flow and aortic growth rate in that cohort. Our FL RF parameter would be sensitive to helical flow, a 3D correlated to in-plane rotational flow. But, in our study, there was no association between FL or combined DAo EL, which is a marker of flow turbulence and vortex formation, and aortic growth rate [19], [26]. Bellala et al. found an association between combined DAo EL and aortic growth rate, but there was no isolation of FL from TL flow [27]. This result was more likely driven by TL flow given the generally higher flow rates and velocities and is less likely representative of FL hemodynamics.

Current treatment strategies for TBAD, both medical and surgical, aim to reduce pressurization of the FL and limit blood flow into the FL overall [8], [11], [12]. Our findings relating FL MV and RF to aortic growth are consistent with the current rationale for clinical management of TBAD. Of note, baseline aortic diameter, a major factor in the current treatment algorithm for TBAD, was not predictive of aortic growth. Longitudinal hemodynamic assessment can stratify TBAD patients within a clinically significant time period, as Fig. 4 demonstrates changes in a patient with only 135 days of follow-up who later required surgical intervention. TBAD patients are already evaluated with serial imaging at 3, 6, and 12 months; longitudinal hemodynamic assessment may be able to meaningfully improve morphological evaluation in TBAD without adding to patient burden.

Another noteworthy takeaway from the results of this study was the difference in behavior between the rTAAD and dnTBAD subgroups. In the rTAAD subgroup, there were positive correlations between changes in FL MV and FL RF with aortic growth, whereas, in the dnTBAD subgroup, there were positive correlations between changes in TL KE and TL MV and growth and the relationships with FL MV and FL RF were not significant. There is growing evidence that these two populations that have historically been evaluated together differ hemodynamically. Jarvis et al. found differences in regional TL and FL flow parameters between rTAAD and dnTBAD patients, and Chu et al. found differences in which hemodynamic parameters were predictive of adverse clinical outcomes and rapid aortic growth between rTAAD and dnTBAD patients. Our results add to these findings, showing that longitudinal hemodynamic evolution differs between these populations, and suggest that rTAAD and dnTBAD may warrant distinct evaluation and risk stratification.

Interestingly, changes in TL rather than FL hemodynamics were predictive of growth in the dnTBAD subgroup. We hypothesize that this could be capturing ongoing ventricular remodeling in response to changes in afterload as a result of evolution of the dissection, which has been seen in response to TBAD and is associated with poor clinical outcomes [28], [29], [30]. However, the absence of correlations with changes in FL hemodynamics in this subgroup could be due to a lack of statistical power from small sample size (n = 13) and only two patients in this subgroup having rapid aortic growth. Larger sample sizes would be needed to confirm these findings.

## 6 Limitations

This study has several limitations. The nature of the longitudinal study may have biased the cohort toward patients with more stable aortas. More patients would need to be enrolled who went on to have adverse clinical outcomes to evaluate the impact of longitudinal hemodynamic changes on rates of descending aorta surgical or endovascular intervention or aorta-related death in addition to aortic growth. Additionally, the observational nature of the study may have limited the ability of the cohort to adequately sample the full range of the natural history of TBAD. A randomized control trial with larger sample sizes would be needed to fully address this.

There are also potential technical limitations to this study. Our sample includes both prospectively ECG-gated and retrospectively gated 4D flow scans due to technological development at our institution, with some patients having sagittal, prospectively ECG-gated scans at baseline and coronal, retrospectively gated scans at follow-up. While spatial resolution did not change, respiratory motion will have differed in the absence of respiratory navigator gating, and there was truncation of late diastole in retrospective scans to match prospective scan duration. Prior work has shown there to be no significant difference between hemodynamic parameters acquired using the two different sequences, but still this may have affected the precision of our results and limited our ability to detect hemodynamic changes during late diastole, or caused underestimation of diastolic retrograde flow [3]. Furthermore, aortic segmentation was performed using a time-averaged method, rather than time-resolved. Using a time-averaged segmentation may have affected hemodynamic parameters due to intimal flap motion [31]. Our flow parameters do not rely on capturing flow at the luminal wall and are likely less affected, but time-resolved segmentation should be considered in future studies. Also, venc was set to capture maximum velocities in the TL. Because flow in the FL is slower, using a single-venc acquisition may have limited the precision of our flow parameters in the FL. Multi-venc acquisitions may better capture slower flow in the FL [32]. Lastly, we did not conduct an interobserver study for pre-processing and manual segmentations because the same method was previously shown to have high similarity, but there is a possibility of manual error in aortic segmentation [1].

## 7 Conclusion

The findings of our study suggest that 4D flow CMR assessment of longitudinal hemodynamic changes provides additional prognostic value for the evaluation of TBAD. Our results also support the separation of rTAAD and dnTBAD in future studies. 4D flow CMR could be integrated into existing imaging protocols to allow for identification of TBAD patients who would benefit from preemptive surgical or endovascular intervention.

## Funding

Work was supported by two grants: 10.13039/100000968American Heart Association 20CDA35310687 and NIH/NHLBI 1R01HL168700-01A1.

## Author contributions

J.S.E. segmented 4D flow datasets, performed statistical analysis, and drafted the manuscript. O.K. segmented 4D flow datasets and recruited patients. E.K.W. and J.B. assisted with the generation of parametric hemodynamic maps. All authors contributed to the conception of the study and interpretation of the data. All authors have read and enthusiastically approved the final manuscript submission.

## Ethics approval and consent

This study uses data collected under protocols approved by the Northwestern University IRB as outlined in the manuscript methods.

## Consent for publication

Not applicable.

## Declaration of competing interests

The authors declare the following financial interests/personal relationships which may be considered as potential competing interests: Bradley Allen reports financial support was provided by the American Heart Association and the National Institutes of Health. Bradley Allen reports a relationship with Third Coast Dynamics that includes board membership, employment, and equity or stocks. Michael Markl reports a relationship with Third Coast Dynamics that includes board membership, employment, and equity or stocks. Bradley Allen reports a relationship with Circle Cardiovascular Imaging Inc. that includes speaking and lecture fees. Co-author serving on the editorial board for the Journal of Cardiovascular Magnetic Resonance, B.A. Co-author serving as president of the Society for Cardiovascular Magnetic Resonance, M.M. The other authors declare that they have no known competing financial interests or personal relationships that could have appeared to influence the work reported in this paper.

## Data Availability

The data that support the findings of this study are available on request from the corresponding author J.S.E. The data are not publicly available due to datasets containing information that could compromise research participant privacy/consent.
