# Supplementary material for: Interval changes in four-dimensional flow-derived in vivo hemodynamics stratify aortic growth in type B aortic dissection patients
Source: J Cardiovasc Magn Reson. 2024 Aug 2;26(2):101078. doi: 10.1016/j.jocmr.2024.101078 (PMC11421234; doi:10.1016/j.jocmr.2024.101078)
Supplement: Supplementary file 1 — Supplementary material [file mmc1.docx]

**Supplementary Table 1:** Energy loss values for baseline and follow-up scans and percent changes in the FL and combined DAo with correlations with aortic growth rate. Variables are reported as median (IQR). FL = false lumen, DAo = descending thoracic aorta, EL_total_ = time-integrated energy loss, EL_mean_ = time-averaged energy loss, nJ = nanojoules, nW = nanowatts, Rho = Spearman’s rho.

| **Overall Cohort (N=32)** | | | | | | | | |
| --- | --- | --- | --- | --- | --- | --- | --- | --- |
| **Parameter** | **Baseline Value** | **Rho** | **P-value** | **Follow-Up Value** | **Percent Change (%)** | **Rho** | **P-value** |  |
| FL EL_total_ (nJ) | 4.12 (3.60) | -0.02 | 0.90 | 2.52 (3.21) | -24.4 (59.2) | 0.09 | 0.63 |  |
| FL EL_mean_ (nW) | 9.87 (11.3) | -0.05 | 0.79 | 6.65 (6.84) | -31.4 (55.8) | -0.15 | 0.41 |  |
| Combined DAo EL_total_ (nJ) | 7.15 (6.45) | -0.25 | 0.16 | 6.02 (4.89) | -13.4 (28.3) | -.24 | 0.19 |  |
| Combined DAo EL_mean_ (nW) | 14.7 (12.5) | -0.32 | 0.08 | 12.6 (9.53) | -19.9 (33.3) | 0.20 | 0.27 |  |
